# Addressing socioeconomic inequalities in obesity: Democratising access to resources for achieving and maintaining a healthy weight

**DOI:** 10.1371/journal.pmed.1003243

**Published:** 2020-07-28

**Authors:** Jean Adams

**Affiliations:** Centre for Diet and Activity Research, MRC Epidemiology Unit, University of Cambridge, Cambridge, United Kingdom

In low-income countries, overweight and obesity are more common in more socioeconomically affluent groups [1]. This pattern flattens and then reverses as country-level income increases. In high-income countries, those living in less affluent circumstances are more likely to experience overweight and obesity. For example, in England, adults living in the most deprived fifth of neighbourhoods are almost twice as likely to be living with obesity (where the prevalence of obesity is 36%) as those living in the least deprived fifth (where the prevalence of obesity is 20%) [2]. These socioeconomic inequalities in unhealthy body weight manifest early in life, with an obvious relationship seen between neighbourhood deprivation and the experience of overweight or obesity in 4- to 5-year-old children in England [3]. As more countries experience epidemiological transitions, this inverse association between socioeconomic position and prevalence of unhealthy weight is becoming more common [1].

The standard energy balance explanation of unhealthy body weight proposes that weight gain occurs, and unhealthy weight is maintained, when energy intake is greater than energy expenditure. The problem of obesity becomes easily framed within this explanation as one of quantity and personal gluttony and laziness: either energy intake is too high, energy expenditure is too low, or both. Applied to the specific case of socioeconomic inequalities in obesity, this framing leads to the proposal that these personal failings are more common in less affluent groups. The obvious solution that can flow is one of personal restraint and discipline, particularly for those living in less affluent circumstances.

A closer look at socioeconomic differences in both dietary and physical activity patterns reveals that these differences may not simply be ones of quantity. Important socioeconomic differences in the quality of both diet and physical activity are becoming clear. For example, there is little evidence of socioeconomic differences in British children’s achievement of international recommendations for 60 minutes of moderate- to vigorous-intensity physical activity per day. But more affluent children do accumulate more of the vigorous-intensity activity that is particularly associated with body weight than their less affluent counterparts, and this appears to be via more participation in organised sport [4]. Similarly, there is little evidence that total dietary energy varies consistently across socioeconomic groups in the United Kingdom, but dietary quality does. Those living in more affluent households eat more fruit and vegetables than those living in less affluent homes, drink fewer sugar-sweetened beverages, and are more likely to consume diets associated with lower cardiovascular risk [5,6].

These findings suggest that we cannot explain socioeconomic inequalities in unhealthy body weight as due to differences in gluttony and laziness, nor view the solution as one of greater personal restraint and discipline. Doing so would be both untrue and unhelpful. Instead, the question becomes one of why there are consistent differences in the quality of diet and physical activity that people living in different circumstances have access to.

Socioeconomic position is often measured in terms of education, income, occupational social class, or neighbourhood circumstances. But the concept captures more than any of these indicators alone. It is about access to resources in their widest sense—certainly financial resources, but also social, physical, cognitive, and other resources.

Recent changes in food practices associated with COVID-19 restrictions highlight how these practices are related to the social and physical resources that people have access to. In April 2020, when most UK schools, restaurants, cafes, and workplaces were closed, and government advice was to ‘stay at home’, half of UK adults reported that they were eating more home-cooked food and less takeaway and fast food than normal [7]. This suggests that longer-term declines in home food preparation [8] may have more to do with changes in predictable time spent at home and the availability of alternative sources of food rather than any widespread loss of cooking skills. And in more ‘normal’ times, these social and physical resources are distinctly socioeconomically patterned. People living in less affluent circumstances are less likely to have predictable working hours, and takeaway outlets are more common in less affluent neighbourhoods [9].

Access to financial resources is a key component of socioeconomic position. Although it is often assumed that absolute destitution is rare in high-income countries, the visit by the United Nations’ Special Rapporteur on Extreme Poverty and Human Rights to the UK in 2019 highlighted this is not the case. His report opens with the estimate that 1.5 million people in the UK were unable to afford basic necessities in 2017 [10].

Recent, but pre-COVID-19, data from the UK indicate that one-fifth to one-quarter of adults experienced food insecurity (i.e., limited or uncertain access to adequate and safe food due to financial constraints) in the previous 12 months [11,12]. Thus, each year, 20%–25% of adults in the UK worry about being able to afford food or skip meals because they cannot afford to buy food. Although it may seem superficially paradoxical, in high-income countries, food insecurity is consistently associated with obesity and poorer dietary quality, particularly in women [13]. This reflects known differences in food prices—healthier foods and diets tend to be more expensive [14]—meaning that under conditions of financial constraint, people turn first to lower-quality, less healthy diets, before sacrificing on absolute energy quantity. The finding of a consistent association between food insecurity and unhealthy body weight further undermines the assumption that obesity is a problem of personal excess and laziness.

Financial constraints may similarly act as a barrier to the organised sports that tend to make up the vigorous physical activity that is most associated with body weight. Many such sports require clothing and equipment to be bought and classes or other facilities to be paid for. Here, too, social and physical resources are important, with less affluent families reporting a lack of time to support their children doing these activities and less actual or perceived access to appropriate facilities [15].

Viewing obesity as a problem of quality, rather than quantity, and understanding socioeconomic position in terms of access to a wide variety of resources lead to the conclusion that socioeconomic inequalities in obesity are due to differential access to the resources required to access high-quality diets and physical activity. Rather than admonishments to the ‘poor’ to eat more prudently or exercise more frequently, the solution to socioeconomic inequalities in obesity presented by this framing is to provide everyone with access to adequate resources to achieve and maintain a healthy body weight. Reshaping fiscal, social, and physical environments to make it easier to access healthier practices—via, for example, planning restrictions on hot food takeaway outlets, taxes on less healthy foods, and subsidies on children’s access to sport—is likely to help. However, the most powerful way to ensure that everyone has adequate access to the resources required to achieve and maintain a healthy weight may be through stronger welfare and employment policies, including higher minimum wages, working hour mandates, and universal basic income [16].
